# “*Echoes from the Dyad*”: Relational Context of Postpartum Depression Risk

**DOI:** 10.3390/jcm15072608

**Published:** 2026-03-29

**Authors:** Wioletta Tuszyńska-Bogucka, Katarzyna Bosowska

**Affiliations:** 1 Faculty of Psychology and Pedagogy, University of Economics and Innovation in Lublin, 20-209 Lublin, Poland; 2 Faculty of Human Sciences, VIZJA University, 01-043 Warsaw, Poland; k.bosowska@vizja.pl

**Keywords:** clinical and health psychology, perinatal trauma, postpartum depression risk, attachment style, state anxiety, partner support, relational context, moderation analysis

## Abstract

**Background:** Postpartum depression (PPD) is a clinically significant condition shaped by emotional regulation processes and close relational contexts. Anxiety is often theorized as a mediating mechanism linking relational vulnerabilities to depressive symptoms, yet empirical findings remain mixed. **Objectives:** This study examined whether state anxiety mediates the association between insecure attachment styles and PPD symptoms or whether its effects depend on relational context, specifically perceived partner support. **Methods:** In this cross-sectional study, a sample of 249 women assessed within 12 months postpartum completed self-report measures of attachment styles in the intimate relationship, state and trait anxiety, perceived partner support, and PPD symptoms. Hypotheses were tested using multiple regression analyses with heteroskedasticity-consistent standard errors, including mediation and moderation models. **Results:** Both anxious–ambivalent and avoidant attachment styles were associated with greater PPD symptom severity. State anxiety was neither an independent predictor nor a mediator of the attachment–PPD relationship. Instead, its association with PPD symptoms was conditional: anxiety was positively related to depressive symptoms only when perceived partner support was insufficient. **Conclusions:** Anxiety may function as a context-sensitive amplifier rather than a universal mechanism of postpartum depressive risk. These findings highlight the potential importance of relational context in understanding emotional vulnerability and depressive symptoms during the postpartum period.

## 1. Introduction

Postpartum depression (PPD) is among the most prevalent mood disorders in the perinatal period, and clinically significant depressive symptoms may persist throughout the first year after childbirth [1,2,3]. In both research and clinical screening, the Edinburgh Postnatal Depression Scale is widely used to screen for elevated risk [4,5]. In the present study, PPD is conceptualized dimensionally as the severity of depressive symptoms assessed within 12 months postpartum, in line with epidemiological and screening conventions [3,6].

The transition to parenthood entails substantial emotional demands, role reorganization, and greater reliance on the quality of the intimate relationship. A robust body of evidence indicates that low relationship satisfaction, partner conflict, and insufficient emotional support are associated with both the onset and maintenance of PPD symptoms [7,8,9]. From a systemic perspective, PPD is therefore increasingly understood not solely as an intrapsychic condition but as a phenomenon arising from relational processes within the partner dyad [10,11].

Attachment theory offers a well-established framework for conceptualizing these relational processes. Adult attachment orientations reflect relatively stable emotion-regulation strategies and expectations about the availability of support in close relationships [12,13]. Under the heightened demands of the perinatal period, insecure attachment—both anxious–ambivalent and avoidant—has been associated with impaired communication of emotional needs, reduced effectiveness of partner support, and increased vulnerability to emotional distress [14,15,16,17]. Accordingly, empirical syntheses consistently identify insecure attachment as a relational risk factor for postpartum depression [15,18].

Anxiety has been proposed as a mechanism linking relational vulnerability with depressive symptoms. Although anxiety and depression frequently co-occur during the perinatal period [19,20], the functional role of anxiety remains theoretically unclear. Attachment-based models frequently propose anxiety as a mediating mechanism linking attachment insecurity with depressive symptomatology [21,22,23]. In contrast, relational and dyadic perspectives emphasize co-regulation within the couple, suggesting that anxiety may acquire clinical significance primarily under conditions of insufficient partner support [10,24,25].

These perspectives imply two distinct mechanisms: (1) mediation, whereby state anxiety transmits the effect of insecure attachment on PPD symptom severity, and (2) moderation, whereby partner support alters the strength or clinical relevance of the association between anxiety and PPD. Although studies have documented associations among attachment insecurity, anxiety, and partner support, previous research has rarely examined these mechanisms simultaneously within a single analytical framework or explicitly contrasted mediation and moderation explanations of postpartum depressive risk.

**Figure 1:**
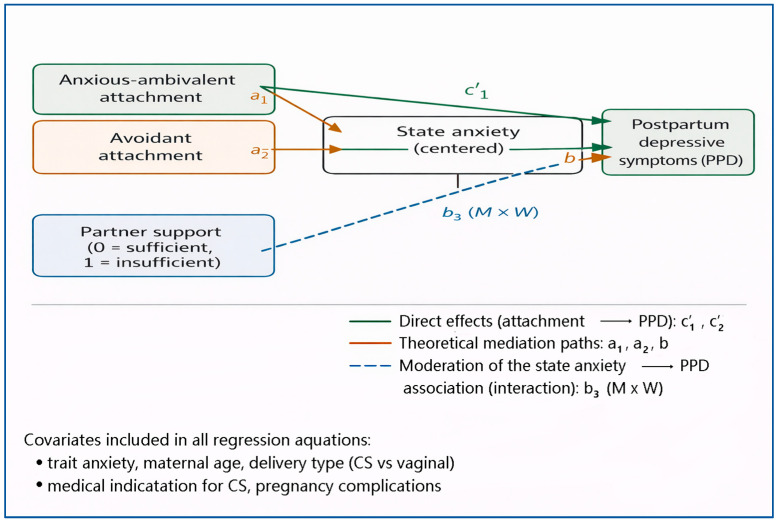
Integrated conceptual framework (nested model).

Accordingly, the present study integrates relational predispositions (attachment styles), a proximal emotional state (state anxiety), and relational context (perceived partner support) in explaining PPD symptom severity, while controlling for trait anxiety and key perinatal factors. By formally testing both mediation and moderation pathways, the study seeks to clarify whether anxiety operates as a general mechanism or as a context-dependent amplifier of depressive risk. Figure 1 presents the integrated analytical framework guiding hypothesis testing, including both the theoretically proposed mediation pathways and the alternative moderation mechanism.

## 2. Materials and Methods

### 2.1. Research Hypotheses

Based on theoretical models of attachment, emotion regulation, and relational functioning, the present study examines how relatively stable relational predispositions interact with current emotional processes during the perinatal period. Functioning within an intimate partnership is conceptualized as a central context shaping emotional regulation and vulnerability to postpartum depressive symptoms.

Main Hypotheses:

**H1** **(main** **effect).** *Insecure attachment styles are associated with greater severity of postpartum depressive symptoms. Both anxious–ambivalent and avoidant attachment are expected to show positive associations with PPD after adjustment for the same set of covariates*.

**H2** **(moderation).** *The association between state anxiety and postpartum depressive symptoms is conditional on perceived partner support. Specifically, higher levels of state anxiety are expected to be associated with greater PPD severity primarily under conditions of insufficient perceived partner support, whereas this association is expected to be weaker or absent when perceived partner support is sufficient*.

**H3** **(mediation).** *State anxiety is hypothesized to mediate the association between insecure attachment styles and postpartum depressive symptoms*.

Additional Analysis:

**A1** **(formal test of H2).** *To facilitate interpretation of the moderation effect specified in H2, simple slope analyses will examine the association between state anxiety and PPD separately across levels of perceived partner support. The anxiety–PPD association is expected to be stronger under conditions of insufficient perceived partner support than when perceived partner support is sufficient*.

### 2.2. Analytical Plan

All analyses were conducted using multiple regression models estimated by ordinary least squares (OLS) and heteroskedasticity-consistent standard errors (HC3). Detailed model specifications are reported in Supplementary Material, Table S2. The analytical strategy followed the theoretical framework presented in Figure 1, with attachment dimensions specified as relational predispositions, state anxiety as a potential mechanism, and postpartum depressive symptom severity as the outcome.

Pre-analytical procedure. Prior to the main regression analyses, Pearson correlations among all study variables were examined and visualized in a correlation graph (Supplementary Material, Figure S1) to provide a descriptive overview of associations within the dataset. Zero-order correlations among study variables are presented in Supplementary Material, Table S1. This step served exclusively for exploratory and diagnostic purposes, including inspection of the general dependency structure and potential multicollinearity. Importantly, these preliminary analyses did not inform the specification of the hypothesis-driven regression models. All predictors and covariates included in the main analyses were selected a priori based on theory and the predefined research hypotheses.

Regression models. The conceptual framework specified insecure attachment dimensions as antecedent relational predispositions, state anxiety as a potential mechanism, and postpartum depressive symptoms as the outcome (Figure 1). Variable distributions and bivariate associations were inspected to assess model assumptions. The main hypotheses were tested using regression models that included anxious–ambivalent and avoidant attachment, state anxiety, perceived partner support, and their interaction. All models were adjusted for trait anxiety, maternal age, delivery type, medical indication for cesarean section, and pregnancy complications.

State anxiety was mean-centered prior to computing the interaction term; all other predictors were entered in their original metric. Multicollinearity diagnostics indicated negligible overlap among predictors (all VIFs ≤ 2.02), supporting stable coefficient estimation. Cook’s distance values remained below conventional thresholds (maximum D = 0.087), indicating no influential observations (Supplementary Material, Table S3).

Indirect effects were examined using bootstrap resampling with 5000 bias-corrected 95% confidence intervals (Supplementary Material, Table S4). Given the cross-sectional design, mediation was interpreted in a statistical rather than temporal sense and therefore does not imply causal ordering among variables. Because both mediation and moderation mechanisms were specified a priori based on competing theoretical accounts, each pathway was formally evaluated. The interaction between centered state anxiety and perceived partner support was further examined using simple slope analyses. Statistical significance was evaluated using two-tailed tests with α = 0.05.

The sample size (N = 249) and the number of predictors included in the final model were consistent with conventional recommendations for multiple regression analyses [26], indicating that effects in the small-to-moderate range (approximately f^2^ ≥ 0.04) were expected to be detectable with adequate statistical power.

### 2.3. Psychological Measures

Postpartum depression. Severity of postpartum depressive symptoms was assessed using the Polish adaptation of the Edinburgh Postnatal Depression Scale (EPDS) [27]. Each item is scored from 0 to 3, yielding a total score range of 0–30, with higher scores indicating greater symptom severity and elevated risk of PPD. The Polish version demonstrates good psychometric properties, with Cronbach’s α coefficients typically exceeding 0.80 in postpartum samples.

Attachment styles. Attachment within the romantic relationship was measured using the Attachment Styles Questionnaire [28], which assesses secure, anxious–ambivalent, and avoidant attachment dimensions (eight items per scale). The scale is grounded in Bowlby’s attachment theory, as extended to adult romantic relationships. It assumes that relatively stable internal working models shape expectations of availability and support from close others, as well as habitual emotion-regulation strategies within intimate partnerships. Accordingly, the questionnaire operationalizes attachment as three relational orientations—secure, anxious–ambivalent, and avoidant—reflecting distinct patterns of closeness, dependence, and affect regulation in close relationships. Higher scores reflect stronger expression of a given attachment style. Previous studies report high reliability for the Polish version, with Cronbach’s α values of approximately 0.91 for secure, 0.78 for anxious–ambivalent, and 0.80 for avoidant attachment [28]. Secure attachment was not included as a predictor, as the analyses focused on insecure attachment dimensions conceptualized as relational risk factors.

Trait and state anxiety. General anxiety vulnerability and current anxiety were assessed using the Polish adaptation of the State-Trait Anxiety Inventory (STAI-T and STAI-S) [29]. Higher scores indicate higher levels of trait or state anxiety. Polish validation studies report very good reliability for both subscales, with α coefficients close to 0.90 (see Supplementary Material, Table S5 for details). Trait anxiety reflects a relatively stable dispositional vulnerability and is therefore appropriately modeled as a covariate to control for baseline anxiety proneness. In contrast, state anxiety represents a situationally activated emotional response and is conceptually suited as a proximal predictor or mediator in process-oriented models of psychopathology [30,31]. State anxiety was examined because it reflects situational emotional reactivity that may be particularly sensitive to the relational context during the postpartum period.

Partner support. Perceived partner support was assessed using a single global item reflecting the overall adequacy of support and dichotomized into sufficient versus insufficient support. The indicator was intended to capture a broad contextual appraisal of partner support rather than to model the multidimensional structure of the construct. This operationalization was selected because partner support was conceptualized as a broad contextual indicator rather than a multidimensional relational construct. For such global evaluations, single-item measures represent a well-established and appropriate alternative to multi-item scales [32]. Global appraisal measures are widely used in psychological and epidemiological research to capture individuals’ overall evaluations of complex constructs, including health status or relationship functioning. Importantly, the present indicator reflects perceived partner support rather than objective partner behavior and should therefore be interpreted as a subjective indicator of relational context within the couple. In postpartum samples, brief assessments additionally reduce respondent burden and the risk of missing data. Although multi-item instruments generally show higher predictive validity for complex latent constructs [33], the present study employed a single-item indicator specifically to operationalize a screening-level relational context for moderation analyses rather than to assess the internal structure of partner support. Perceived partner support was coded as 0 = sufficient and 1 = insufficient.

Covariates. Maternal age, trait anxiety, delivery type, medical indication for cesarean section, and pregnancy complications were included as control variables in all analyses.

Perinatal window. Eligibility required that the most recent delivery occurred within the preceding 12 months. Although diagnostic classifications define the postpartum period more narrowly for episode onset, epidemiological evidence indicates that both risk and persistence of depressive symptoms extend across the first year after childbirth [2,3,6,20,34] and may persist even longer [35]. Trajectory studies further identify women with elevated or newly emerging symptoms throughout this period [6,20,35]. A summary of relevant trajectory findings is provided in Supplementary Material, Table S5B. Accordingly, EPDS scores obtained up to 12 months postpartum were treated as indicators of symptom severity. The cross-sectional design did not stratify analyses by time since childbirth, which limits precision when generalizing findings to specific postpartum phases. Early postpartum months are often more strongly associated with biological and obstetric factors, whereas later months are increasingly shaped by relational and psychosocial processes. This heterogeneity should be considered when interpreting the results. Consistent with the study aims, the focus was placed on symptom severity rather than diagnostic classification, supporting the use of a broader postpartum window.

### 2.4. Participants and Recruitment

The sample consisted of 249 women assessed within 12 months postpartum (mean age = 32.96 years, SD = 4.13; range = 22–41). Cesarean delivery was reported by 53.8% of participants; among these cases, 70.7% reported medical indications for cesarean delivery. Pregnancy complications were reported by 25.7% of the total sample. The majority of participants reported sufficient perceived partner support, whereas 16.1% reported insufficient partner support. Mean EPDS scores indicated moderate depressive symptom severity (M = 9.49, SD = 4.81), along with average levels of state anxiety (M = 45.60, SD = 10.34) and trait anxiety (M = 46.61, SD = 8.81). Mean scores for anxious–ambivalent and avoidant attachment were relatively low within the possible scale ranges. Detailed sample characteristics are presented in Table 1.

Recruitment, which included online sampling within a single geographic region, may have introduced selection bias, as participation could have been influenced by symptom severity or health-seeking behavior.

Procedure. The study was conducted in 2025 in the Silesian Voivodeship, Poland. Data were collected either online or in paper-and-pencil format during individual meetings; in both cases, participants completed identical self-report questionnaires without interviewer assistance. In both conditions, participants received full information about the study aims and procedures prior to providing their responses.

### 2.5. Summary of Descriptive Statistics and Correlations

PPD symptoms were positively correlated with state anxiety, anxious–ambivalent attachment, and avoidant attachment. Neither attachment dimension was significantly related to state anxiety, indicating that attachment insecurity co-occurred with depressive symptoms but not with elevated situational anxiety at the bivariate level of analysis. Insufficient perceived partner support was associated with both insecure attachment dimensions, whereas its bivariate association with PPD symptoms was nonsignificant. The strongest association was observed between anxious–ambivalent and avoidant attachment.

Although model specification was guided by theory, the observed pattern of bivariate associations is more consistent with a moderation framework than with a mediation pathway at the descriptive level. Specifically, the absence of bivariate associations between attachment dimensions and state anxiety argues against a simple mediation mechanism and is consistent with a moderation model in which the effect of state anxiety on PPD depends on perceived partner support. Detailed descriptive statistics and correlation coefficients are presented in Table 2.

## 3. Results

### 3.1. Main Effects and Associations Among Attachment Dimensions, State Anxiety, and Postpartum Depressive Symptoms

Both anxious–ambivalent and avoidant attachment were significant predictors of postpartum depressive symptoms in the covariate-adjusted regression model, with effects of comparable magnitude (Table 3). No statistically significant difference in effect magnitude was observed between the two attachment dimensions. Perceived partner support was also a statistically significant predictor (B = −1.685, *p* = 0.044). However, because a significant interaction between state anxiety and perceived partner support was observed (B = 0.177, *p* = 0.012), this coefficient reflects the group difference at the mean level of centered state anxiety and should not be interpreted as an unconditional main effect. The association between state anxiety and postpartum depressive symptoms was therefore examined using simple slope analyses. The full model explained 18.8% of the variance in postpartum depressive symptoms (R^2^ = 0.188, adjusted R^2^ = 0.154), F(10, 238) = 5.45, *p* < 0.001.

### 3.2. Moderation Analysis

**Figure 2:**
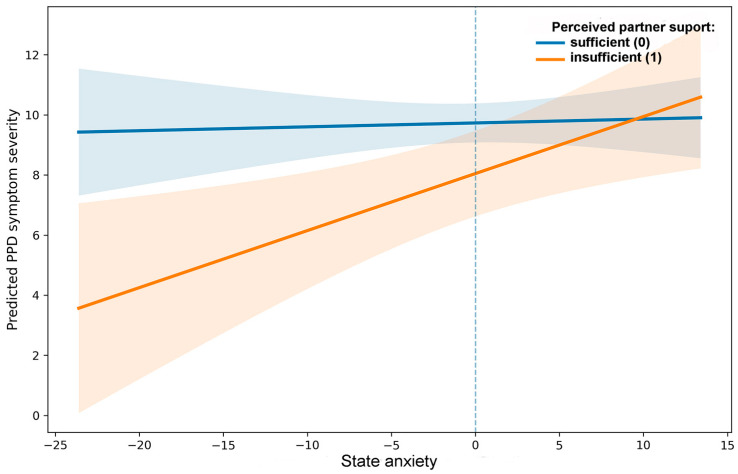
Moderating effect of perceived partner support on the association between state anxiety and PPD symptoms. The slope for insufficient perceived partner support was significant (*p* = 0.007), whereas the slope for sufficient support was nonsignificant (*p* = 0.758). Shaded areas represent 95% confidence intervals.

A moderation model examined whether perceived partner support modified the association between state anxiety and postpartum depressive symptoms (Figure 2). The main effect of state anxiety was not statistically significant (B = 0.013, *p* = 0.769). The coefficient for perceived partner support was negative (B = −1.685, *p* = 0.044); however, because the predictor entered the model together with the interaction term, this coefficient reflects the group difference at the mean level of centered state anxiety and does not represent an unconditional main effect.

A significant interaction between state anxiety and perceived partner support was observed (B = 0.177, *p* = 0.012), indicating that the association between anxiety and postpartum depressive symptoms varied as a function of partner support. The interaction accounted for additional variance beyond the main effects (ΔR^2^ = 0.019). The standardized interaction coefficient was β = 0.15 (Supplementary Material, Table S8).

Simple slope analyses (SSA) indicated that state anxiety was positively associated with postpartum depressive symptoms among women reporting insufficient perceived partner support (B = 0.190, *p* = 0.007). In contrast, under sufficient perceived partner support, the association was nonsignificant (B = 0.013, *p* = 0.758). The slope under sufficient support did not differ from zero, indicating no statistical evidence for an association between anxiety and PPD in that subgroup. Detailed SSA estimates with 95% confidence intervals are reported in Supplementary Material, Table S8, and stratified estimates in Table S9.

**Figure 3:**
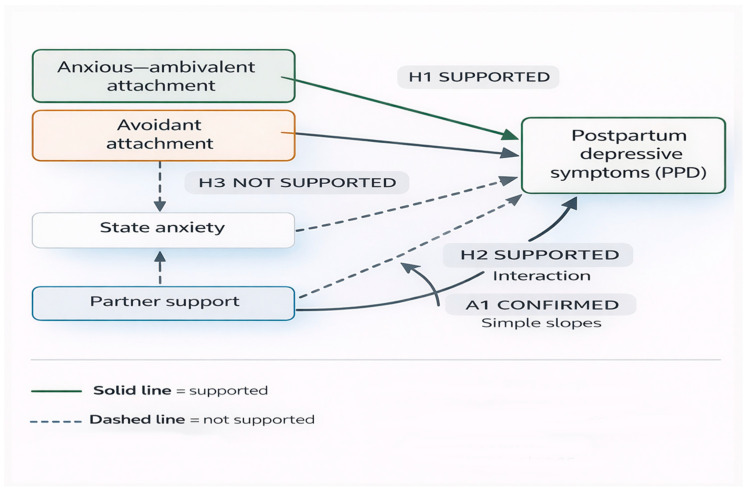
Results-based summary of the nested analytical model.

Figure 3 illustrates how the tested hypotheses relate to one another within the analytical framework. Insecure attachment styles are positioned as antecedent relational vulnerabilities showing direct associations with postpartum depressive symptoms. State anxiety was examined as a potential mediating mechanism but did not transmit the effect of attachment on depressive symptoms. Instead, perceived partner support altered the association between anxiety and depressive symptoms: elevated state anxiety was associated with higher PPD symptom severity only when perceived partner support was insufficient, whereas under sufficient support this association was not observed. The diagram therefore depicts a conditional pattern in which relational context is associated with whether emotional distress translates into depressive symptoms.

### 3.3. Hypothesis Testing

The study hypotheses were evaluated using the regression, moderation, and bootstrap procedures described above.

**H1.**  *Insecure attachment styles and PPD symptoms. In covariate-adjusted regression models controlling for trait anxiety, maternal age, delivery type, medical indication for cesarean section, and pregnancy complications, both anxious–ambivalent and avoidant attachment were significantly and positively associated with postpartum depressive symptom severity (Table 3). Thus, H1 was supported*.

**H2.**  *Conditional association between state anxiety and PPD symptoms. Regression analyses indicated that the association between state anxiety and postpartum depressive symptoms varied as a function of perceived partner support. The interaction between state anxiety and perceived partner support was statistically significant, supporting the conditional nature of the anxiety–PPD association (Figure 2). Accordingly, H2 was supported*.

**H3.**  *Mediation via state anxiety. Bootstrapped mediation analyses indicated that the indirect effects of anxious–ambivalent and avoidant attachment on postpartum depressive symptoms via state anxiety were not statistically significant, as the confidence intervals included zero (Supplementary Material, Table S4). The direct associations between attachment dimensions and PPD symptoms remained significant after inclusion of state anxiety in the models. Therefore, H3 was not supported*.

**Additional** **Analysis** **(A1).** *Formal test of moderation. The moderation hypothesis was evaluated by estimating the interaction between state anxiety and perceived partner support. Simple slope analyses indicated that state anxiety was positively associated with postpartum depressive symptoms under conditions of insufficient perceived partner support, whereas under sufficient perceived partner support the slope did not differ from zero. These findings support and further specify the moderation pattern proposed in H2 by demonstrating differential anxiety–PPD slopes across levels of perceived partner support*.

Figure 3 presents a graphical summary of the tested mediation pathways and the empirically supported moderation effect.

The diagram summarizes the empirical status of the tested hypotheses concerning associations among insecure attachment, state anxiety, perceived partner support, and postpartum depressive symptoms. The association between state anxiety and postpartum depressive symptoms is depicted as conditional on perceived partner support, consistent with the significant interaction and simple slope analyses.

## 4. Discussion

Overview of main findings. The present study examined relational and emotional determinants of postpartum depressive symptom severity in the context of perceived partner support. Insecure attachment styles and perceived partner support emerged as central factors associated with PPD severity [7,14,36], whereas the role of state anxiety was conditional rather than uniform. These findings support relational models in which postpartum depressive symptoms emerge from configurations of emotional vulnerability and interpersonal context rather than from any single predictor examined in isolation [10,11,37].

Epidemiological syntheses demonstrate substantial heterogeneity in the comorbidity of anxiety and depression across the perinatal period [38,39,40]. Evidence that clinically relevant depressive symptoms may occur throughout the first postpartum year further underscores the importance of context-sensitive screening and intervention [3,6,35]. Relationship quality—particularly marital satisfaction—consistently emerges as a major risk domain for postpartum depression [7,8,9]. Recent umbrella reviews converge in identifying interpersonal functioning and perceived partner support as robust and modifiable risk domains across healthcare systems [41,42].

Attachment style and PPD. Consistent with H1, insecure attachment styles were associated with greater postpartum depressive symptom severity. Adult attachment theory provides a framework for understanding how internal working models shape emotion regulation and expectations within close relationships [12,13]. Empirical studies show that insecure attachment increases vulnerability to postpartum depression and improves identification of women at risk [14,15,18,22,23,43,44]. Longitudinal and trajectory-based research further indicates that attachment-related vulnerabilities are linked to persistent anxiety–depression profiles and to deterioration in relationship quality during the transition to parenthood [17,45,46].

State anxiety beyond mediation models. Contrary to H3 but consistent with H2, state anxiety neither independently predicted postpartum depressive symptom severity nor mediated the association between attachment and PPD. Although anxiety and depression frequently co-occur in the perinatal period [11,18,19], their association appears to be context-dependent once relational factors are taken into account [7,36,47]. The present findings suggest that state anxiety reflects situational emotional strain rather than a universal pathway to postpartum depression.

Importantly, the absence of mediation does not diminish the clinical relevance of anxiety. Anxiety-related distress appears to acquire depressive significance primarily under conditions of relational vulnerability. This interpretation is consistent with cohort evidence showing that anxiety predicts later PPD predominantly in subgroups exposed to additional stressors [48] and with broader reviews conceptualizing anxiety as one component of a multifactorial psychosocial constellation [39].

Conceptual contribution. The standardized interaction effect (β = 0.15) was comparable in magnitude to the direct effects of avoidant attachment (β = 0.16) and perceived partner support (β = −0.13), and slightly exceeded that of trait anxiety (β = 0.12). This pattern suggests that postpartum depressive vulnerability may be better understood as arising from configurations of emotional and relational factors rather than from any single predictor considered in isolation. Although the incremental variance explained by the interaction was modest (ΔR^2^ ≈ 0.02), effect sizes of this magnitude are typical for psychosocial moderators in observational research. The overall model explained 18.8% of the variance in depressive symptom severity (R^2^ = 0.188), indicating a meaningful—though not exhaustive—explanatory framework. Together, these findings suggest that anxiety is associated with elevated depressive risk primarily in contexts of insufficient partner support. Additional stratified analyses supported this interpretation: the association between state anxiety and PPD symptoms was significant only among women reporting insufficient partner support (Supplementary Material, Table S9). Rather than functioning as a general mediator, anxiety appears to operate as a context-sensitive correlate of vulnerability within the relational environment. In this context, the moderating effect observed in the present study likely reflects the perceived relational environment within the couple rather than objectively measured partner behavior. Thus, the present findings may reflect not the mere presence of supportive behaviors but the psychological meaning attributed to relational support within the dyad. This interpretation aligns with epidemiological and trajectory-based evidence showing that anxiety exerts its strongest effects when embedded in contexts of relational or socioeconomic adversity [40,46], as well as with large-scale syntheses emphasizing partner and family factors as central components of perinatal risk constellations [41,42].

An additional point concerns the use of perceived partner support as the relational-context variable in the present study. In psychological research, perceived support represents a theoretically meaningful construct rather than merely a proxy for objectively enacted supportive behaviors. From a psychological perspective, emotional regulation and well-being depend primarily on the perceived responsiveness of close relationships rather than on the objective frequency of supportive behaviors, which may explain why perceived partner support in the present study functioned as a contextual moderator rather than a direct predictor of depressive symptoms [49,50]. Emotional adjustment depends not only on whether supportive actions occur but on whether support is experienced as available, responsive, and adequate within the relationship. This perspective is consistent with the pattern observed in the present study. Partner support did not emerge as a strong independent predictor of PPD symptom severity but instead moderated the association between anxiety and depressive symptoms. Such a pattern is theoretically compatible with models of relational regulation, according to which the psychological impact of emotional distress depends on the perceived relational climate in which it occurs. In this sense, perceived partner support may function as an indicator of the dyadic context that shapes whether anxiety remains a transient emotional state or becomes associated with more persistent depressive symptomatology. Similar patterns have been observed in perinatal research, where women’s perceptions of partner support have been shown to predict postpartum emotional adjustment more consistently than structural indicators of support or the frequency of supportive behaviors [51,52]. From a methodological perspective, the use of a single-item indicator of perceived partner support should be interpreted in relation to the nature of the construct being assessed. The aim of the measure in the present study was to capture a global appraisal of relational support within the dyad rather than the multidimensional structure of supportive behaviors. Methodological research indicates that single-item measures may be appropriate when the objective is to assess global evaluations of clearly defined constructs rather than their internal structure [32,33]. Within this framework, a global assessment may adequately reflect the respondent’s perception of relational responsiveness, which is the dimension most directly relevant for emotional regulation and depressive vulnerability. 

Clinical implications. Screening for postpartum depression should incorporate assessment of partner relationship quality and perceived support. Elevated anxiety occurring in contexts of insufficient perceived partner support may signal a conditionally higher-risk profile [47,53]. Population-based studies increasingly recommend combined screening for anxiety and depression while explicitly considering relational stressors [39,40].

Interventions focused solely on anxiety reduction may be insufficient when relational functioning is not addressed. Systematic reviews indicate that strengthening partner involvement and dyadic coping enhances maternal mental-health outcomes [7,8,36]. Emerging evidence further highlights the protective role of perceived partner support during childbirth and early postpartum adjustment, particularly among women with pre-existing vulnerabilities [54].

Study limitations. The cross-sectional design and reliance on self-report measures preclude causal inference and may introduce shared-method variance. Indirect paths were therefore interpreted as statistical consistency rather than evidence of temporal ordering. Longitudinal and dyadic studies are needed to capture dynamic processes within intimate relationships [10,17,45]. Repeated assessments across the first postpartum year are warranted, given evidence that symptom trajectories vary over time [35,46]. Although no formal a priori power analysis for moderation was conducted, the final sample size (N = 249) aligns with conventional recommendations for regression models with multiple predictors [26]. The observed interaction effect was small in magnitude (f^2^ = 0.023), which is typical for psychosocial moderation effects in observational research. Finally, although the conceptual framework adopts a dyadic perspective, the present study relied exclusively on maternal reports. Perceived partner support was additionally assessed using a single global item reflecting perceived adequacy of support. Although single-item indicators are commonly used for global relational evaluations, they do not capture the multidimensional structure of perceived partner support and may be partly influenced by the respondent’s current emotional state; accordingly, the present indicator should be interpreted as a global appraisal of relational support rather than as a detailed measure of supportive behaviors. However, in the present dataset perceived partner support showed no meaningful association with depressive symptom severity, suggesting that this indicator was unlikely to simply reflect current depressive mood. This design reflects the study’s focus on maternal risk identification and screening-relevant mechanisms, for which perceived partner support from the mother’s perspective is the most clinically informative indicator.

Practical meaning. State anxiety alone is insufficient for identifying women at risk for postpartum depression. From a clinical perspective, the relevant question is whether anxiety occurs in a context where partner support is perceived as insufficient. When perceived partner support is present, anxiety shows little association with depressive symptoms; when it is perceived as insufficient, the association between anxiety and PPD becomes stronger. Risk assessment and early intervention should therefore target the combination of emotional distress and relational context rather than anxiety in isolation.

Take-home message. Postpartum depressive risk emerges when emotional distress is embedded in an unfavorable relational context. Insecure attachment styles constitute enduring relational vulnerabilities, whereas perceived partner support appears to shape whether anxiety becomes clinically consequential. Thus, models that incorporate relational context provide a more psychologically meaningful account of postpartum depression than approaches focusing solely on individual emotional symptoms.

## Figures and Tables

**Table 1:** Characteristics of the study sample (N = 249).

Characteristic	Value
Maternal age, years—M (SD)	32.96 (4.13)
Cesarean delivery—%	53.8
Medical indication among CS—%	70.7
Pregnancy complications—%	25.7
Insufficient perceived partner support—%	16.1
Trait anxiety—M (SD)	46.61 (8.81)

**Table 2:** Descriptive statistics and correlations among main variables.

Variable	M	SD	1	2	3	4	5
1. State anxiety	45.60	10.34	-	0.181 **	−0.047	−0.005	0.044
2. Postpartum depressive symptoms	9.49	4.81	0.181 **	-	0.278 ***	0.275 ***	0.024
3. Anxious–ambivalent attachment	21.30	10.65	−0.047	0.278 ***	-	0.520 ***	0.254 ***
4. Avoidant attachment	18.13	9.41	−0.005	0.275 ***	0.520 ***	-	0.358 ***
5. Perceived partner support	–	–	0.044	0.024	0.254 ***	0.358 ***	-

Note. Values are Pearson correlation coefficients (r) shown in the lower triangle; for the binary variable partner support, coefficients represent point-biserial correlations., ** *p* < 0.01, *** *p* < 0.001.

**Table 3:** Multiple regression predicting PPD symptoms.

Predictor	B	SE (HC3)	*p*
Intercept	0.680	3.654	0.852
Anxious–ambivalent attachment	0.092	0.036	0.011
Avoidant attachment	0.115	0.048	0.017
State anxiety (centered)	0.013	0.044	0.769
Perceived partner support (1 = insufficient)	−1.685	0.838	0.044
State anxiety × Perceived partner support	0.177	0.071	0.012
Trait anxiety	0.095	0.050	0.058
Maternal age	0.024	0.076	0.749
Delivery type (cesarean)	−0.613	0.628	0.329
Medical indication for CS	−0.022	0.637	0.972
Pregnancy complications	0.479	0.722	0.507

Note. Model adjusted for trait anxiety, maternal age, delivery type, medical indication for cesarean section, and pregnancy complications.

## Data Availability

The data presented in this study are not publicly available due to ethical restrictions and the sensitive nature of the information related to women’s mental health in the postpartum period. Anonymized data may be made available from the corresponding author upon reasonable request and subject to appropriate ethical approval.

## Supplementary Materials

The following supporting information can be downloaded at: https://www.mdpi.com/article/10.3390/jcm15072608/s1, Figure S1: Graph-based correlation network of candidate predictors; Table S1: Zero-order correlations; Table S2: Heteroskedasticity diagnostics (baseline model); Table S3: Multicollinearity and influence diagnostics; Table S4: Bootstrap indirect effects (attachment → anxiety → PPD); Table S5A: Measures and psychometric characteristics; Table S5B: Operationalization of postpartum depression (literature review); Table S6: Regression model—anxious attachment; Table S7: Regression model—avoidant attachment; Table S8: Simple slopes and interaction effect (anxiety × partner support); Table S9: Stratified associations (state anxiety–PPD by partner support).

## Abbreviations

The following abbreviations are used in this manuscript:

PPD	Postpartum Depression
EPDS	Edinburgh Postnatal Depression Scale
STAI-T	State–Trait Anxiety Inventory–Trait
STAI-S	State–Trait Anxiety Inventory–State
OLS	Ordinary Least Squares
HC3	Heteroskedasticity-consistent standard errors
VIF	Variance Inflation Factor
SSA	Simple Slope Analyses
