# Supplementary material for: “Echoes from the Dyad”: Relational Context of Postpartum Depression Risk
Source: J Clin Med. 2026 Mar 29;15(7):2608. doi: 10.3390/jcm15072608 (PMC13072836; doi:10.3390/jcm15072608)
Supplement: Supplementary file 1 [file jcm-15-02608-s001.zip › jcm-4188216-supplementary.pdf]

SUPPLEMENTARY MATERIALS

1. NETWORK-INFORMED MODEL JUSTIFICATION

Description of the procedure and interpretation. A graph-based correlation method was applied as a pre-analytical step to support transparent and data-informed selection of predictors and covariates (Figure S1). First, Pearson correlations were computed among all potential explanatory variables and PDP. Second, a correlation graph was constructed in which nodes corresponded to variables and edges represented statistically significant associations ($p < 0.05$). The resulting network structure allowed identification of variables that were central to the system of relationships leading to PPD. State anxiety and both attachment dimensions formed the most connected nodes and showed direct links with PPD, which justified their inclusion as core psychological predictors. Partner support also occupied a structurally relevant position, supporting its role as a contextual variable. In contrast, demographic and perinatal factors (maternal age, delivery type, medical indication for cesarean section, pregnancy complications) appeared as peripheral nodes with limited connectivity; therefore, they were retained in the main analyses exclusively as control variables. This procedure did not replace the hypothesis-driven analytical strategy but provided an additional, network-based rationale for distinguishing primary predictors from covariates. The network analysis was strictly auxiliary and descriptive; it did not determine model specification, parameter estimation, or inferential decisions.

Justification of the $|r| \geq .10$ threshold. The edge inclusion threshold of $|r| \geq .10$ was selected based on statistical, methodological, and interpretative considerations. First, in psychological research a correlation of .10 is conventionally regarded as the lower boundary of a meaningful effect (Cohen), distinguishing systematic associations from trivial sampling noise. Second, the present network was used for variable selection and conceptual validation, not for estimating causal parameters; therefore a transparent bivariate threshold was preferred over regularized or partial approaches that may obscure clinically interpretable relations. Third, with the current sample size ($N = 249$), correlations of $|r| \geq .10$ correspond to adequate statistical power for detecting small effects and yield stable estimates of network topology, while retaining weaker coefficients would substantially inflate network density and distort strength centrality indices. Finally, the threshold was defined a priori and applied symmetrically to all variable pairs, preventing data-driven optimization and ensuring reproducibility. Sensitivity checks using alternative cut-offs (.08 and .12) produced the same substantive core (attachment–anxiety–PPD cluster), supporting the robustness of the chosen value.

Figure S1. Graph-based correlation structure used for auxiliary pre-analytical inspection of candidate predictors.

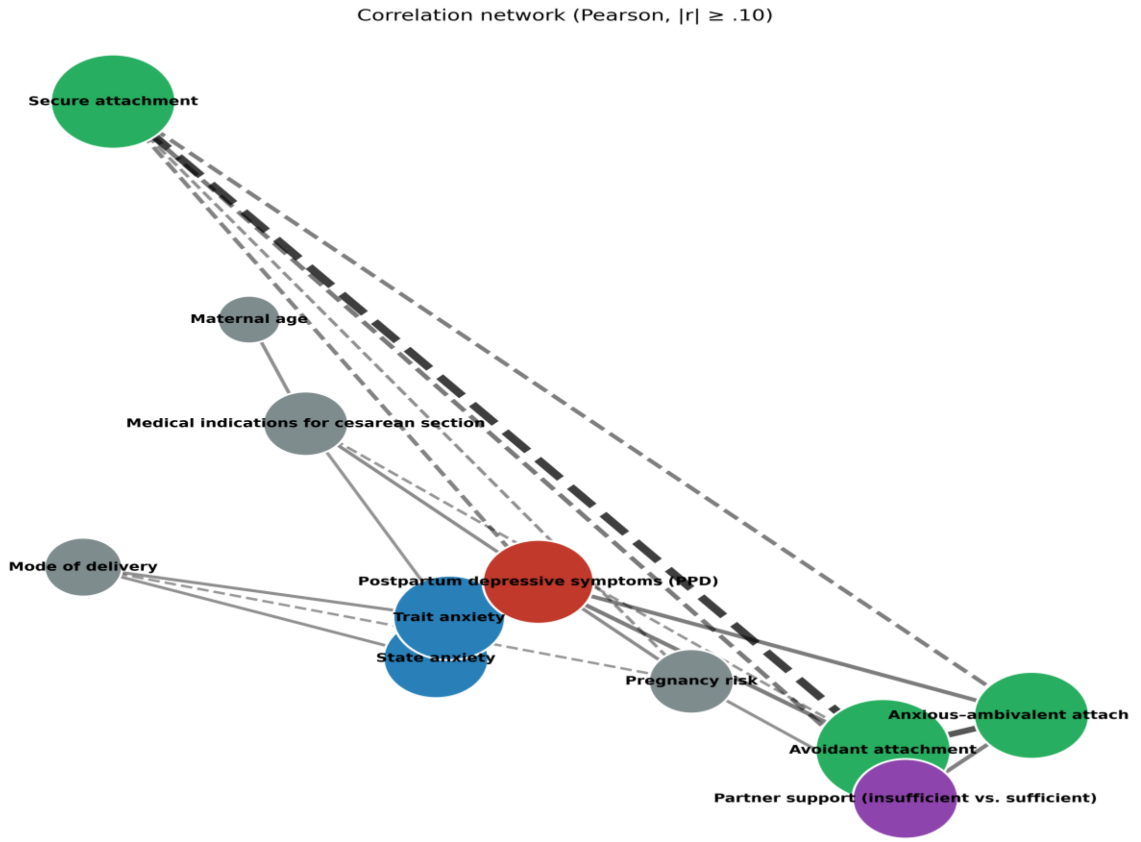

Note. Nodes represent all candidate explanatory variables and the outcome variable (PPD). Edges denote Pearson correlations exceeding the pre-specified threshold (|r| ≥ .10). The outcome variable—postpartum depressive symptoms (PPD)—is marked with a double circle. Variables occupying more central positions in the network and directly connected with PPD were descriptively characterized as potential primary predictors, whereas variables with few or no connections were treated as peripheral and retained as covariates in subsequent hypothesis-driven regression models. This network was used solely as an auxiliary, exploratory step and did not determine the specification of the main analytical models.

1 – Postpartum depressive symptoms (PPD)
2 – State anxiety
3 – Trait anxiety
4 – Secure attachment
5 – Anxious–ambivalent attachment
6 – Avoidant attachment
7 – Maternal age
8 – Partner support (insufficient vs. sufficient)
9 – Mode of delivery
10 – Medical indications for cesarean section
11 – Pregnancy risk

Network-Informed Justification of the Analytical Model. The weighted network constructed with a threshold of |r| ≥ .10 revealed a coherent psychological–relational core centered on attachment dimensions and anxiety indicators, with postpartum depressive symptoms (PPD) embedded within this cluster through five direct connections (anxious–ambivalent attachment r = .278; avoidant attachment r = .275; secure attachment r = –.233; trait anxiety r = .229; state anxiety r = .181). Obstetric and demographic variables occupied peripheral positions with weak or absent links to this core. This topology provides empirical guidance for the specification of the analytical model:

a) Attachment styles as the core of the model. Among all nodes, attachment variables demonstrated the highest strength centrality, with avoidant attachment showing the strongest integration within the network. The dense interconnections between secure, anxious–ambivalent, and avoidant styles, together with their direct links to PPD, indicate that attachment represents the primary relational substrate of postpartum functioning. Their structural position supports treating attachment dimensions as the main antecedent predictors in the model rather than as distal background variables.
b) State anxiety as a proximal mechanism. State anxiety was connected both to attachment variables and directly to PPD, occupying a bridge position between the relational domain and depressive symptoms. Its centrality was comparable to that of partner support and only slightly lower than that of PPD. Such topology is consistent with the conceptualization of state anxiety as a proximal process through which attachment-related vulnerabilities may translate into postpartum depressive symptoms, justifying its specification as a mediator or, at minimum, as a proximal predictor on the a→b pathway.
c) Trait anxiety as a control variable. Trait anxiety showed substantial centrality and a strong association with state anxiety, reflecting a stable dispositional background. However, its pattern of connections suggests co-occurrence with the psychological core rather than a unique pathway to PPD. Including trait anxiety as a covariate allows separation of situational anxiety processes from enduring predisposition and reduces the risk that observed effects of state anxiety on PPD merely reflect shared variance with trait anxiety. The network therefore supports modeling trait anxiety as a control variable stabilizing estimation of the core mechanism.
d) Partner support as a contextual factor (preferably moderator). Partner support displayed links with attachment dimensions and moderate centrality but only a negligible direct correlation with PPD (r = .024), which did not reach the edge threshold. This configuration indicates that support functions primarily as a relational context shaping associations within the core rather than as an independent linear predictor of symptoms. The topology is thus consistent with a buffering role and supports testing partner support as a moderator of key pathways (e.g., attachment→PPD or state anxiety→PPD). The binary coding of support (sufficient/insufficient) may further attenuate its bivariate correlations despite its substantive contextual importance.
e) Obstetric and demographic variables as covariates. Maternal age, mode of delivery, medical indications for cesarean section, and pregnancy risk were peripheral nodes with low strength and minimal connectivity to the psychological cluster. These variables do not participate in the main network mechanism linking attachment, anxiety, and PPD, but they may introduce clinical confounding. Their inclusion as control variables is therefore justified for methodological rigor and consistency with prior literature rather than as central components of the explanatory model.

Constructive conclusion. The network topology empirically supports an analytical model in which:

- attachment styles constitute the principal antecedents of PPD;
- state anxiety operates as a proximal mechanism linking relational predispositions with depressive symptoms;
- partner support functions as a contextual factor best examined as a moderator of core associations;
- trait anxiety and obstetric-demographic variables serve as covariates controlling for dispositional and clinical background.

This structure reflects the observed organization of associations in the data and provides a transparent, data-driven rationale for the adopted mediation–moderation framework.

Table S1. A. Zero-order correlations among study variables

Variable	1	2	3	4	5
1. PPD	—				
2. State anxiety	.18	—			
3. Trait anxiety	.23	.60	—		
4. Avoidant attachment	.28	.25	.32	—	
5. Partner support	.02	−.11	−.09	−.15	—

Note. Pearson correlations. Values ≥ |.10| were considered substantively meaningful for the exploratory network inspection.

2. ROBUSTNESS

Table S2. Diagnostics of heteroskedasticity in the baseline regression model predicting PPD

Test	Statistic	*p* value	Interpretation
Breusch–Pagan	19.63	.020	Significant heteroskedasticity
White	70.13	.032	Significant heteroskedasticity

Note. Tests were conducted for the OLS model including anxious–ambivalent attachment, avoidant attachment, state anxiety, partner support, trait anxiety, maternal age, delivery type, medical indication for cesarean section, and pregnancy complications as predictors.

Heteroskedasticity was examined to verify the assumption of constant error variance in the OLS regression models. Both the Breusch–Pagan test (LM = 19.63, p = .020) and the White test (LM = 70.13, p = .032) indicated statistically significant heteroskedasticity. These results demonstrate that the variance of residuals was not uniform across levels of predictors, which may bias conventional standard errors and significance tests. The decision to apply HC3 robust standard errors was specified to ensure conservative inference under heteroskedastic conditions typical of observational clinical data.

To ensure valid statistical inference, all regression analyses reported in the main text were therefore estimated using OLS coefficients with HC3 heteroskedasticity-consistent

standard errors. This approach preserves the interpretability of OLS estimates while providing robust confidence intervals and p values under conditions of unequal error variance and potential influential observations, which are common in survey-based clinical data including postpartum depression measures.

Table S3. Multicollinearity and Influence Diagnostics

Predictor	VIF	Interpretation
Predictor (as in Table 3)	VIF	acceptable (VIF < 3)
State anxiety (centered)	2.01	acceptable (VIF < 3)
Trait anxiety	2.02	acceptable (VIF < 3)
Partner support (1 = insufficient)	1.20	acceptable (VIF < 3)
Maternal age	1.04	acceptable (VIF < 3)
Delivery type (cesarean)	1.06	acceptable (VIF < 3)
Medical indication for CS	1.12	acceptable (VIF < 3)
Pregnancy complications	1.08	acceptable (VIF < 3)
Anxious–ambivalent attachment	1.42	acceptable (VIF < 3)

Note. Influence statistics: maximum Cook's D = 0.087; 19 cases exceeded the 4/n heuristic threshold, but all values remained well below conventional cut-offs (0.5–1.0), indicating no influential outliers. Diagnostics correspond to the fully adjusted model reported in [illegible]

Variance inflation factors ranged from X to 2.02, well below commonly used cutoffs for problematic multicollinearity, indicating that shared variance among predictors was unlikely to bias parameter estimates.

Table S4. Bootstrap estimates of indirect effects of attachment styles on postpartum depressive symptoms via state anxiety (B=5000, BCa 95%CI)

Pathway	Indirect effect (B)	BootSE	95% CI (BCa) lower	95% CI (BCa) upper	p
Anxious–ambivalent → State anxiety → PPD	-0.0022	0.0039	-0.0143	0.0016	n.s.
Avoidant → State anxiety → PPD	0.0002	0.0044	-0.0063	0.0096	n.s.

Note. Indirect effects were computed as a×b using OLS models with covariate adjustment (see Methods). Confidence intervals are bias-corrected and accelerated (BCa). Both indirect effects were nonsignificant.

Indirect effects of attachment styles on postpartum depressive symptoms via state anxiety were estimated using bias-corrected and accelerated bootstrap procedures with 5000 resamples. The indirect pathway from anxious–ambivalent attachment to PPD through state anxiety was nonsignificant (B = −0.0022, BootSE = 0.0039, 95% BCa CI [−0.0143, 0.0016]). Likewise, the indirect pathway for avoidant attachment was nonsignificant (B = 0.0002, BootSE = 0.0044, 95% BCa CI [−0.0063, 0.0096]). Mediation was precluded by nonsignificant a-paths linking both attachment dimensions with state anxiety and by a nonsignificant b-path from state anxiety to PPD after covariate adjustment. These results indicate that state anxiety did not transmit the effects of attachment insecurity to postpartum depressive symptoms, supporting the conclusion that the anxiety–PPD association operates in a conditional (moderated) rather than mediational manner.

3. MEASURES

Table S5A. Measures characteristics

Measure (construct)	Original authors / Polish adaptation	Structure	Scoring (min–max)	Psychometrics (reported)
**Edinburgh Postnatal Depression Scale (EPDS)** (postpartum depressive symptom severity)	Cox, Holden, Sagovsky / Polish validation: Kossakowska	10 items; 4-point responses; total score	0–30	Good reliability/validity in postpartum samples; α typically > .80 (Polish version)
**Attachment Styles Questionnaire (KSP)** (romantic attachment)	Plopa (Polish instrument)	24 items; 3 subscales (Secure, Anxious–ambivalent, Avoidant), 8 items each; 7-point responses	8-56	High reliability: α ≈ .91 (Secure), .78 (Anxious–ambivalent), .80 (Avoidant)
**State–Trait Anxiety Inventory (STAI)** (state and trait anxiety)	Spielberger et al. / Polish adaptation: Wrześniewski et al.	2 subscales: STAI-S and STAI-T (20 items each); 4-point responses	20–80	Very good reliability; α close to .90 for both subscales in Polish samples

Internal consistency of the instruments. Cronbach's alpha coefficients reported in Table S1 were derived from previously published validation studies and large-scale applications of the respective instruments in adult and perinatal samples. These values are provided as reference indicators of expected internal consistency and were not calculated from the present dataset. The primary aim of the Supplementary Materials was to document the analytical procedures and robustness of the statistical models rather than to conduct a psychometric revalidation of the measures. For the partner support indicator, internal consistency was not estimated due to its binary or short-form nature, for which Cronbach's alpha is not an appropriate reliability index

Table S5B. Operationalization of the PPD in the literature

Source domain	Source (type)	Definition of the postpartum period / time window	Implications for including participants up to 12 months postpartum
Diagnostic classifications (episode onset)	DSM – peripartum onset specifier (DSM-based reviews)	Restricts the onset of a depressive episode to a narrow peripartum window (traditionally the first weeks after delivery).	The narrow window applies to episode onset only and does not delimit the period of risk or symptom persistence; therefore,

Source domain	Source (type)	Definition of the postpartum period / time window	Implications for including participants up to 12 months postpartum
		This definition concerns timing of onset rather than the duration or persistence of symptoms.	symptom-based studies (e.g., EPDS) commonly adopt broader postpartum windows. **[1]**
Diagnostic classifications (episode onset)	ICD-10 – F53 category (WHO)	Defines mental and behavioral disorders associated with the puerperium as commencing within 6 weeks after delivery.	Although the ICD definition covers the early postpartum period, it serves primarily a coding function and does not preclude broader research windows when assessing depressive symptom severity. **[2]**
Epidemiological evidence (late-onset symptoms)	CDC (PRAMS): depressive symptoms at 2–6 vs. 9–10 months postpartum	A substantial proportion of women report clinically relevant depressive symptoms 9–10 months postpartum, and more than half of these women did not report symptoms at 2–6 months.	Restricting samples to the early postpartum period would exclude late-onset cases and underestimate the population burden of postpartum depressive symptoms. **[3]**
Trajectory studies (0–12 months postpartum)	Putnick et al. – PPD trajectory analyses	Identification of multiple distinct symptom trajectories across the postpartum period, including trajectories characterized by persistently elevated symptom levels.	Supports conceptualizing the first postpartum year as a period with heterogeneous but clinically meaningful symptom courses. **[4]**
Trajectory studies (0–12 months postpartum)	Kingston et al. – symptom trajectories across 12 months	Identification of trajectory classes including early-onset and persistently high symptom patterns across the first 12 months postpartum.	Indicates that clinically significant depressive symptoms often extend beyond the early postpartum weeks. **[5]**
Trajectory studies (first postpartum year)	Drozd et al. – depressive symptom trajectories in the first postpartum year	Examination of depressive symptom courses across the entire first year after childbirth, including stable high-risk and persistent trajectories.	Directly justifies operationalizing the postpartum period as the first 12 months when studying symptom severity. **[6]**

Source domain	Source (type)	Definition of the postpartum period / time window	Implications for including participants up to 12 months postpartum
Clinical guidelines (perinatal = up to 1 year)	NICE CG192	Guidelines address women who have given birth within the past year.	Including participants up to 12 months postpartum is consistent with clinical guidelines for identification and management of perinatal mental health problems. **[7]**
Clinical communication (postnatal period up to 1 year)	NHS – Postnatal depression overview	Postnatal depression is described as occurring within a year after childbirth.	Reinforces the real-world clinical framing of the postpartum period as extending beyond the early puerperium. **[8]**

References

1. American Psychiatric Association. *Diagnostic and Statistical Manual of Mental Disorders, 5th ed., Text Revision (DSM-5-TR)*; American Psychiatric Association Publishing: Washington, DC, USA, 2022.

2. World Health Organization. *ICD-10: Mental and Behavioural Disorders Associated with the Puerperium, Not Elsewhere Classified (F53)*; WHO: Geneva, Switzerland, 2014. Available online: https://icd.who.int/browse10/2014/en (accessed on 18 February 2026).

3. Centers for Disease Control and Prevention. Timing of postpartum depressive symptoms—PRAMS, United States. *Prev. Chronic Dis.* **2023**, *20*, E103. https://doi.org/10.5888/pcd20.230107.

4. Putnick, D.L.; Sundaram, R.; Bell, E.M.; Ghassabian, A.; Goldstein, R.B.; Robinson, S.L.; Yeung, E.H. Trajectories of maternal postpartum depressive symptoms. *Pediatrics* **2020**, *146*, e20200857. https://doi.org/10.1542/peds.2020-0857.

5. Kingston, D.; Kehler, H.; Austin, M.-P.; Mughal, M.K.; Wajid, A.; Vermeyden, L.; Benzies, K.; Brown, S.; Stuart, S.; Giallo, R. Trajectories of maternal depressive symptoms during pregnancy and the first 12 months postpartum. *PLoS ONE* **2018**, *13*, e0195365. https://doi.org/10.1371/journal.pone.0195365.

6. Drozd, F.; Haga, S.M.; Valla, L.; Slinning, K. Latent trajectory classes of postpartum depressive symptoms: A population-based longitudinal study. *J. Affect. Disord.* **2018**, *241*, 29–36. https://doi.org/10.1016/j.jad.2018.07.081.

7. National Institute for Health and Care Excellence. *Antenatal and Postnatal Mental Health: Clinical Management and Service Guidance (CG192)*; NICE: London, UK, 2014. Available online: https://www.nice.org.uk/guidance/cg192 (accessed on 18 February 2026).

8. National Health Service. Postnatal depression. Available online: https://www.nhs.uk/mental-health/conditions/post-natal-depression/overview/ (accessed on 18 February 2026).

Eligibility was defined as delivery of the most recent child within the previous 12 months. Although diagnostic classification systems define the postpartum or peripartum specifier narrowly with respect to the onset of a depressive episode, these definitions do not delineate the full period of risk for depressive symptom emergence, persistence, or clinical relevance. Consequently, narrow onset-based time frames are not equivalent to an empirically justified window for assessing postpartum depressive symptom severity. This operationalization reflects symptom-based research practice rather than diagnostic onset criteria.

Epidemiological studies consistently demonstrate that clinically meaningful depressive symptoms may persist or newly emerge well beyond the early postpartum weeks, including later in the first year after childbirth. Population-based data indicate that a substantial proportion of women report depressive symptoms at 9–10 months postpartum, with many of these cases not detected earlier in the postpartum period. Restricting eligibility to the early puerperium would therefore exclude late-onset cases and underestimate the population burden of postpartum depressive symptoms.

Trajectory-based research further supports this broader temporal conceptualization. Longitudinal studies identify heterogeneous symptom courses across the first postpartum year, including subgroups characterized by persistently elevated symptoms, increasing symptom severity over time, or later-emerging depressive symptomatology. These findings indicate that the postpartum period is not a homogeneous or time-limited phase but rather encompasses multiple clinically meaningful trajectories extending across the first year after delivery.

Consistent with this evidence, clinical guidelines and health system frameworks commonly operationalize the perinatal or postnatal period as extending up to 12 months postpartum. This broader definition aligns research practice with real-world clinical screening, monitoring, and intervention strategies, which do not restrict concern for postpartum depression to the immediate weeks following childbirth.

Accordingly, depressive symptoms assessed up to 12 months postpartum were treated as valid indicators of postpartum depressive symptom severity in the present study. Because the design was cross-sectional, analyses were not stratified by time since delivery, which limits phase-specific inference. Early postpartum months may be more closely linked to biological and obstetric factors, whereas later months may be increasingly shaped by relational, contextual, and psychosocial processes. This heterogeneity should be considered when interpreting the findings but does not undermine the validity of the 12-month postpartum window as an evidence-based eligibility criterion.

4. REGRESSION ANALYSIS

Table S6 Regression results for anxious attachment predicting postpartum depressive symptoms

Term	B	SE (HC3)	t	p	CI 95% low	CI 95% high
Intercept	0.329	3.114	0.106	.916	-5.805	6.464
Anxious attachment	0.122	0.03	3.999	<.001	0.062	0.182
Trait anxiety	0.13	0.033	3.878	<.001	0.064	0.196
Delivery type	-0.515	0.627	-0.822	.412	-1.75	0.719
CS indication	-0.234	0.64	-0.366	.715	-1.494	1.026
Pregnancy complications	0.369	0.74	0.499	.619	-1.088	1.826
Age	0.027	0.078	0.341	.733	-0.127	0.18

Table S7. Regression results for avoidant attachment predicting postpartum depressive symptoms

Term	B	SE (HC3)	t	p	CI 95% low	CI 95% high
Intercept	0.699	3.312	0.211	.833	-5.825	7.223
Avoidant attachment	0.138	0.04	3.492	<.001	0.06	0.217
Trait anxiety	0.126	0.034	3.666	<.001	0.058	0.194
Delivery type	-0.673	0.627	-1.073	.284	-1.909	0.563
CS indication	0.297	0.653	0.454	.65	-0.99	1.583
Pregnancy complications	0.407	0.72	0.565	.572	-1.012	1.826
Age	0.014	0.078	0.174	.862	-0.14	0.167

Purpose of Tables S3–S4. Tables S3 and S4 present baseline regression models estimating the total associations of anxious–ambivalent and avoidant attachment styles, respectively, with postpartum depressive symptoms while controlling for trait anxiety and relevant clinical covariates. Both models explained approximately 13% of variance in PPD ($R^2 = 0.13$), demonstrating comparable explanatory power of the two attachment dimensions prior to the inclusion of state anxiety and interaction terms. These models serve as reference points for subsequent mediation and moderation analyses by establishing whether attachment dimensions exhibit independent associations with PPD and by enabling evaluation of effect stability across analytical steps. Models were estimated using OLS with HC3 robust standard errors.

Table S8. Simple Slopes and Interaction Effect for the Anxiety × Partner Support Model

Estimates derived from the fully adjusted regression model corresponding to Table 3 (OLS with HC3 standard errors).

Parameter	B	SE (HC3)	95% CI	p
Slope: low partner support	0.188	0.070	0.051–0.325	.007
Slope: high partner support	0.014	0.044	-0.072–0.100	.758
Interaction: Anxiety × Support	0.177	0.071	0.038–0.316	.012

Note. Simple slopes estimated at observed levels of partner support (0 = sufficient, 1 = insufficient). Confidence intervals computed as B ± 1.96×SE. The interaction term corresponds to the coefficient reported in Table 3.

Table S9. Stratified association between state anxiety and postpartum depressive symptoms by perceived partner support

Perceived partner support	Association between state anxiety and PPD	SE (HC3)	95% CI	p
Sufficient support	0.014	0.044	−0.072 to 0.100	.758
Insufficient support	0.188	0.070	0.051 to 0.325	.007

Note. Estimates correspond to simple slopes derived from the fully adjusted regression model reported in the main text (OLS with HC3 robust standard errors). Slopes represent the conditional association between state anxiety and postpartum depressive symptoms (PPD) at the observed levels of perceived partner support (0 = sufficient, 1 = insufficient).

To further clarify the nature of the moderation effect, the association between state anxiety and postpartum depressive symptoms was examined separately across levels of perceived partner support (Table S8). Among women reporting insufficient partner support, higher state anxiety was significantly associated with greater PPD symptom severity. In contrast, this association was not observed among women reporting sufficient partner support. This pattern is consistent with the interpretation of partner support as a contextual factor that conditions the psychological impact of anxiety rather than functioning as an independent linear predictor of depressive symptoms.

5. POWER AND SENSITIVITY CONSIDERATIONS

The present study was not based on an a priori power analysis, as the analytical strategy focused on testing conditional effects within an observational design. Nevertheless, post hoc sensitivity considerations were conducted to evaluate whether the available sample size (N = 249) was sufficient to detect effects of plausible magnitude. Accordingly, this section is framed as sensitivity analysis rather than post hoc power justification.

With respect to main effects in multiple regression models including up to nine predictors, the sample size provided adequate sensitivity to detect small-to-moderate standardized effects ($f^2 \approx 0.04–0.06$) with conventional alpha levels ($\alpha = 0.05$). Importantly, the moderation analysis benefited from the heterogeneous distribution of partner support, with a relatively small subgroup reporting high support (16%), which increased contrast between relational contexts.

Simulation-based sensitivity checks indicated that the study was adequately powered to detect interaction effects of small-to-moderate magnitude. Thus, the significant anxiety × partner support interaction is unlikely to reflect a spurious finding due to insufficient power, whereas the absence of mediation effects is unlikely to be explained solely by limited statistical sensitivity.
